# Evaluating Policresulen for Disbudding Dairy Calves: A Two-Part Study on Calf Welfare and Consumer Perceptions

**DOI:** 10.3390/ani15202977

**Published:** 2025-10-14

**Authors:** Tássia Barrera de Paula e Silva, Luís Henrique Rodrigues Silva, Marina Madureira Ferreira, Lorraina Stefanie Moreira de Paula, Alex Lopes da Silva, Marcos Inácio Marcondes, João Henrique Cardoso Costa, Polyana Pizzi Rotta

**Affiliations:** 1 Department of Animal Science, Universidade Federal de Viçosa Minas Gerais, Viçosa 36570-900, Brazil; tassia.silva@ufv.br (T.B.d.P.e.S.); luis.henrique@ufv.br (L.H.R.S.); mm3373@cornell.edu (M.M.F.); lorraina.paula@gmail.com (L.S.M.d.P.); alex.lopes@ufv.br (A.L.d.S.); 2 William H. Miner Agricultural Research Institute, Chazy, NY 12921, USA; mmarcondes@whminer.com; 3 Department of Animal Sciences, University of Vermont, Burlington, VT 05405, USA; joao.costa@uvm.edu

**Keywords:** animal welfare, dairy calves, disbudding, policresulen

## Abstract

**Simple Summary:**

In this study, two methods of disbudding dairy calves were compared: the traditional hot iron technique and a novel approach using the drug policresulen. Policresulen, an acid compound derived from formaldehyde and used in veterinary medicine for wound healing, was effective in inhibiting horn bud growth during the rearing phase and provided moderate pain relief. However, partial regrowth of horns was observed during the heifer phase, with regrowth reaching about a third of the size of horns of non-dehorned calves. A consumer perception survey showed that people without farming experience considered the policresulen method to be less invasive and less labor intensive than conventional technique, with hot iron. Consumers were also willing to pay a premium for dairy products from farms using the medical disbudding method.

**Abstract:**

Disbudding is a common practice on dairy farms, with the hot iron method (HID) widely used, though it causes considerable pain if no analgesia is provided. This study included two experiments. In Experiment 1, an alternative method using policresulen (POD) was evaluated in 24 Holstein calves randomly assigned to either POD or HID at 21 ± 2 days of age. Calves in the POD group received 0.2 mL of 36% policresulen per horn bud, while those in the HID group were fully cauterized. The cornual nerve was blocked with 5 mL of 2% lidocaine in both treatments, and all calves received meloxicam (0.5 mg/kg body weight) for three days post-procedure. Calves treated with POD exhibited fewer pain-related behaviors, such as scratching the horn buds, rubbing against objects, and head shaking, and showed faster horn bud regression. However, 12-month observations revealed that 9 of 12 POD-treated calves showed horn regrowth, indicating limited long-term effectiveness. Experiment 2 assessed consumer perceptions through a questionnaire and video with 236 participants. Participants with farming experience were more familiar with disbudding and preferred HID. In contrast, individuals with less agricultural contact demonstrated a greater willingness to pay for products from farms implementing animal welfare practices, with 76% favoring POD. Overall, participants experienced in agribusiness prioritized technical knowledge and practicality, while others valued animal welfare and were willing to pay higher prices for welfare-friendly practices.

## 1. Introduction

With the intensification of dairy farming, cattle are increasingly kept in confined spaces with high stocking densities where the presence of horns can hinder management and jeopardize safety. Horns pose a risk to humans and animals, particularly as cattle establish social hierarchies through aggressive interactions [1].

Horns are bony projections of the frontal bone covered by a keratinized sheath. The horn buds usually appear in the first days of life and remain attached to the skull until around 2 months of age. During this time, the corium—the tissue that supports horn development—begins to fuse the horn bud to the skull. The optimum time window for preventing horn growth is therefore within the first two months of life, when the corium is destroyed by disbudding. After this stage of development, the horn is completely fused to the skull and requires more invasive surgical removal, which is more painful and stressful for the animal [2].

Hot iron cauterization remains the most widely used method of disbudding on dairy farms and is generally effective when used correctly [3]. However, it is known to cause considerable pain and results in open wounds that can take 6 to 13 weeks to heal, potentially leading to ongoing discomfort [4]. The use of caustic paste is one of the suggested alternatives to hot-iron disbudding. It is a strongly alkaline substance that causes third-degree chemical burns in the germinal tissue of the horn bud. However, the literature reports long healing times, acute inflammatory pain, pain-related behavioral changes (such as head shaking and prostration), and even horn regrowth [5]. Furthermore, highlight that the duration, nature, and intensity of pain caused by chemical burns are likely different from those caused by thermal burns [6].

Policresulen has recently attracted interest as a potential alternative disbudding method for calves, particularly within the Alta Cria program [7]—one of the largest calf and heifer data collection programs in the world, with over 207,000 animals participating. Policresulen, a compound of sulfonic acid and formaldehyde, is frequently used in human medicine to heal wounds [8], remove warts [9] and treat diseases of the urogenital tract [10]. In veterinary medicine, it has been used to treat disorders of the reproductive tract in horses [11] and to promote wound and hoof healing in dairy cows [12]. Due to its high acidity, policresulen acts by protein coagulation, promoting selective necrosis of devitalized tissue and stimulating the tissue regeneration process. This protein coagulation ability can facilitate the degradation of horn buds in calves, which consist of immature keratinized epithelium characterized by proteins that are still disorganized and not fully structured [13].

The constant search for less invasive and less painful methods is rooted in the need to improve animal welfare in dairy production systems, mainly due to the growing public concern for animal welfare. This concern can have a direct impact on the consumption and purchase intentions of products from farms that adopt practices to improve animal health and welfare [14]. With the aim of adopting practices that cause less discomfort when disbudding dairy calves, this study aimed to evaluate the effectiveness of policresulen as an alternative to the traditional method of hot iron disbudding and to compare the pain and discomfort caused to calves by both methods. The second aim was to determine whether consumers’ purchasing intentions for dairy products are influenced by farm welfare practices. It can be hypothesized that policresulen would be an effective and less painful alternative to disbudding with hot iron and that consumers would evaluate this method more positively.

## 2. Materials and Methods

### 2.1. Study 1—Disbudding Techniques

The trial was conducted at the Dairy Cattle Teaching, Research and Development Unit of the University of Viçosa, Viçosa, Minas Gerais, Brazil.

Twenty-four Holstein dairy calves were blocked by sex and randomly assigned to one of two disbudding treatments: policresulen disbudding (POD) or hot iron disbudding (HID), with 12 calves per group (2 males and 10 females). At birth, calves were weighed and housed individually. Colostrum was administered at 10% of body weight with a Brix value of 25° within the first 2 h of life, followed by a second feeding (5% of body weight) of colostrum of similar quality 8 h after birth. Water was provided ad libitum. The calves were fed milk twice daily according to the following schedule: 3 L of transition milk per feeding until 5 days of age, 3 L of raw milk per feeding from 6 to 30 days of age, 4 L per feeding from 31 to 60 days of age, and 3 L per feeding until weaning at 90 days of age. A starter concentrate (22% crude protein and 35% starch in dry matter basis) consisting of soybean meal, ground maize, a mineral mixture and a flavor enhancer was offered ad libitum from birth to weaning. From 40 days of age, Coast Cross hay was offered ad libitum. After weaning, which occurred at 90 days of age, calves were placed in group pens and fed a mixed total ration consisting of corn silage and a concentrate of soybean meal, ground maize, and mineral-vitamins premix (17% crude protein and 30% starch in dry matter basis) once daily.

Disbudding was performed at 21 ± 2 days of age. The calves were restrained in the right lateral position and the corns were cut, first the horn bud on one side, followed by the treatment, and then the cutting and treatment of the second horn bud. The cornual nerve of the horn buds was blocked with 5 mL of 2% lidocaine hydrochloride in both treatments 5 min before disbudding. In POD calves, 0.2 mL of policresulen (Albocresil^®^-360 mg/g, Takeda Pharma LTDA, Jaguariuna, SP, Brazil) was injected into the center of each horn bud using a 30 × 0.8 mm needle. In HID calves, the horn bud was cauterized with a gas-heated iron (Jarejo^®^, Mariana, MG, Brazil) after local anesthesia. All calves received meloxicam (0.5 mg/kg body weight, intramuscularly) for three consecutive days after the procedure.

Calf behavior was observed by three evaluators from 12 h prior to disbudding until 48 h afterwards by previously trained observers using an adapted ethogram [5]. Recorded behaviors included head shaking, scratching the horn area with a limb, scratching the horn on objects, vocalizations and eating. Wound healing was assessed using standardized photographs taken twice a week for 10 weeks after disbudding. The images were taken with a semi-professional Nikon^®^ camera 15 cm from the calf’s head and used for visual comparisons over time. To measure the regression of the horn buds, a caliper (Mitutoyo Sul Americana LTDA^®^, Jundiaí, São Paulo, Brazil) was used, a measuring instrument designed to measure the distance between two opposite surfaces, internal or external, and measurements were taken on days 0, 10, 20, 30, and 60 after disbudding.

Blood samples were taken at 0 (before disbudding), 15 min, 1, 12, 24 h, and 3 and 7 days after disbudding to determine serum cortisol concentrations. Samples were collected via jugular venipuncture and stored in 5 mL non-anticoagulating vacuum tubes with gel, centrifuged at 1500× *g* for 15 min and stored at −20 °C. Cortisol concentrations were measured by chemiluminescence and expressed in nanomoles per liter of serum [15]. Heart rate and respiratory rate were recorded at 0, 0.5, 1, 1.5, 2, 4, 6 and 24 h after disbudding. Heart rate was measured by counting heartbeats auscultated with a veterinary stethoscope for 1 min. Respiratory rate was measured by observing the expansion and relaxation of the thoracic cavity for 1 min.

Growth performance was assessed by average daily gain (ADG). The ADG was calculated from the monthly body weight measurements by dividing the weight change by the number of days since disbudding. Calves were weighed 1, 30, 60, and 90 days after birth using an electronic scale. Calves were weighed on three consecutive days and the average was used to minimize variation due to gut filling.

Data were analyzed using the PROC GLIMMIX procedure from SAS (Release 3.1.0, SAS, OnDemand for Academics, SAS Institute Inc., Cary, NC, USA). For each outcome variable, the d − 1 measurement was tested as a covariate and removed from the model if it was not significant (*p* > 0.05). The main and interaction effects of disbudding method (POD vs. HID) and time were included, with sex serving as a random blocking factor. Time was treated as a repeated measure in the model according to the following structure:

$$Y_{ijkl}=\;\mu \;+\;M_{i}+{\;\delta }_{ij}\;+\;T_{k}+{\left(M\times T\right)}_{ik}\;+S_{l}+\;\varepsilon_{ijkl}$$

where: *Y_ijkl_* = observation *ijk*; *µ* = the overall mean; *M_i_* = fixed effect of method *i*; *δ_ij_* = random error with mean 0 and variance *σ_δ*2, the variance between animals within the method, and it is equal to the covariance between repeated measurements within animals; *T_k_* = fixed effect of time *k*; (*M* × *T*)*_ik_* = fixed effect of interaction between method *i* and time *k*; *S_l_* is the random effect of calf sex *l*, and *ε_ijkl_* = random error with mean 0 and variance *σ*2, the variance between measurements within animals. Fifteen variance-covariance structures were tested for each response variable. Next, the variance-covariance structure that provided the best fit based on the lowest Akaike information criterion. The observations with externally studentized residuals greater than |2.5| were first checked to ensure that it was not a recording error. After checking and assessing that it was not a recording error, the observations were considered outliers and consequently excluded from the dataset. The analysis of possible outliers was only carried out once for each result variable to avoid erroneous outliers in the loop. The least square means were considered different if *p* < 0.05.

### 2.2. Study 2—Dairy Consumer Perceptions of Calf Disbudding Methods

A video was produced to demonstrate the two disbudding techniques investigated in this study. Short descriptions of each procedure accompanied the video, which was uploaded to YouTube (https://www.youtube.com/watch?v=fRtyvn69nR0&t=12s (accessed on 27 January 2024). A seven-item questionnaire was developed to assess participants’ perceptions of disbudding, covering the following topics: (1) occupation, (2) prior knowledge of disbudding, (3) advocacy of disbudding, (4) perceived ease of performing each technique, (5) perceived invasiveness, (6) consumers of dairy products, (7) willingness to pay a premium price for dairy products from farms that use welfare-friendly disbudding methods, and (8) preference regarding which disbudding method justifies paying a higher price.

The questionnaire and video link were distributed via social media, such as WhatsApp and Instagram, to approximately 1000 randomly selected individuals. A total of 236 responses were received. Based on the participants’ professions and their knowledge of disbudding, people were divided into two groups: those with experience in the agricultural sector and those without. This categorization allowed for a more comprehensive interpretation of participants’ familiarity with and perceptions of the disbudding methods presented.

Descriptive analyses were performed using logistic regression, with odds ratios (OR) estimated relative to the HID method as the reference category. Statistical analyses were performed using the PROC GLIMMIX procedure in SAS (Release 3.1.0, SAS OnDemand for Academics, SAS Institute Inc., Cary, NC, USA), and significance was indicated at *p* < 0.05.

## 3. Results

### 3.1. Study 1—Disbudding Techniques

#### 3.1.1. Behavioral Observations and Wound Healing

**Figure 1:**
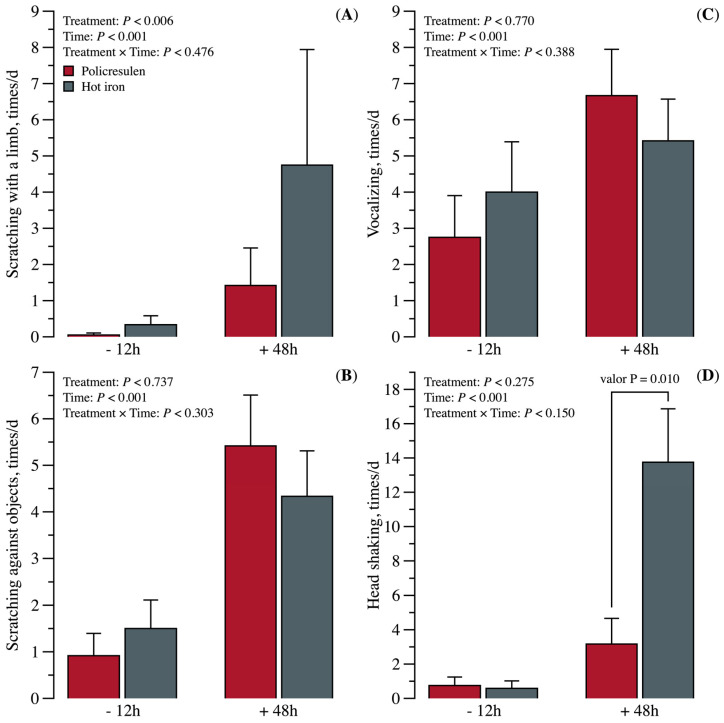
Behavioral responses of calves before (−12 h) and after disbudding (+48 h) using either policresulen method or the hot iron method. (**A**) Scratching behavior with the limb; (**B**) Behavior while scratching an object; (**C**) Vocalizing behavior; (**D**) Head shaking.

There were no interaction effects (*p* > 0.05) between treatment and time for scratching with a limb, scratching against objects, vocalizing, and head shaking (Figure 1). However, there was an effect (*p* < 0.006) of treatment on scratching with a limb, with a higher value observed in the HID group. There were also effects (*p* < 0.001) of time on scratching with a limb, scratching against objects, and head shaking, with greater values at 48 h of evaluation.

**Figure 2:**
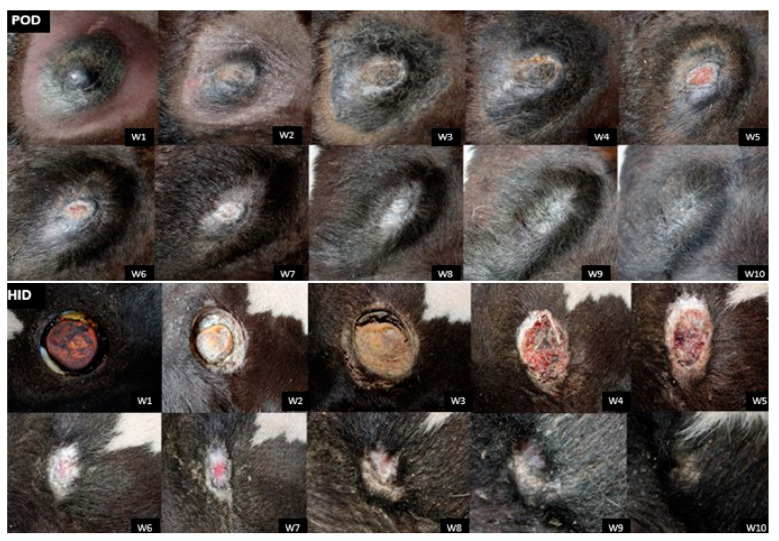
Weekly photographic monitoring of the complete epithelialization process in the horn region of calves subjected to disbudding using either policresulen method (POD) or the hot iron method (HID).

Complete epithelialization of the horn region was observed in both groups and on both horn buds 10 weeks after disbudding (Figure 2).

**Figure 3:**
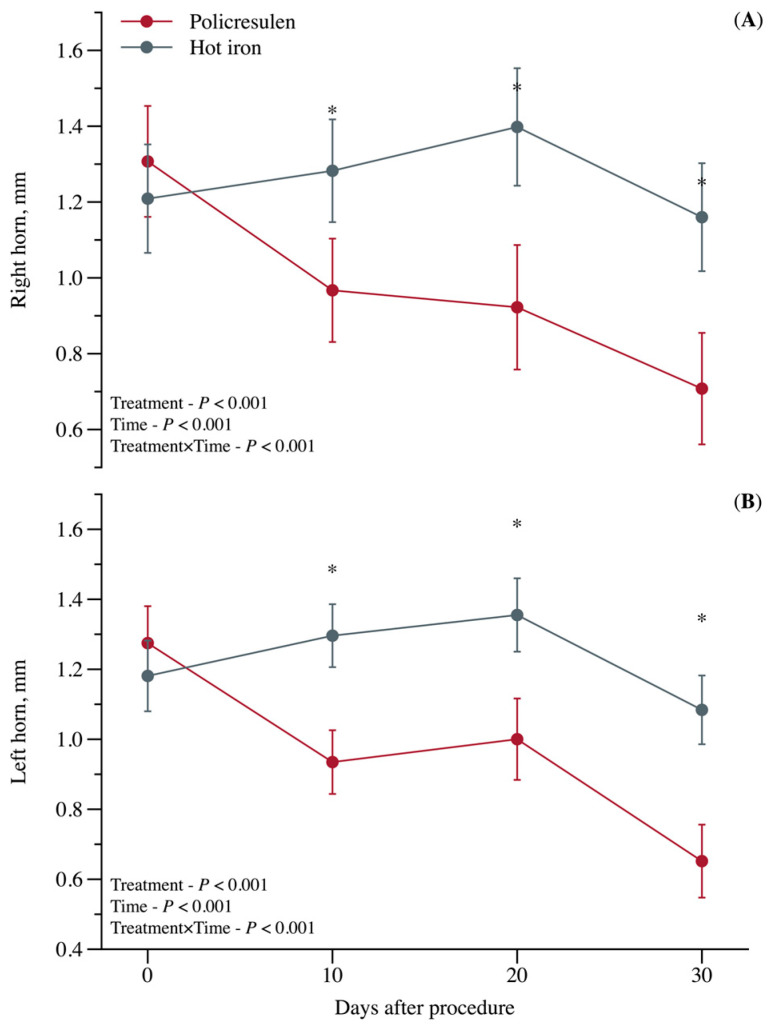
Temporal regression (days) of the corneal button diameter in calves subjected to disbudding using either policresulen method or the hot iron method. (**A**) Right horn; (**B**) Left horn. * Points of statistical differences between the two treatments.

There was also an interaction between treatment and time (*p* < 0.001; Figure 3) on horn bud circumferences. At d0, no difference was observed between treatments. However, for 10, 20, and 30 d after the procedure, a greater reduction in circumferences was observed in the POD group than in the calves in the HID group.

#### 3.1.2. Physiological Responses

**Figure 4:**
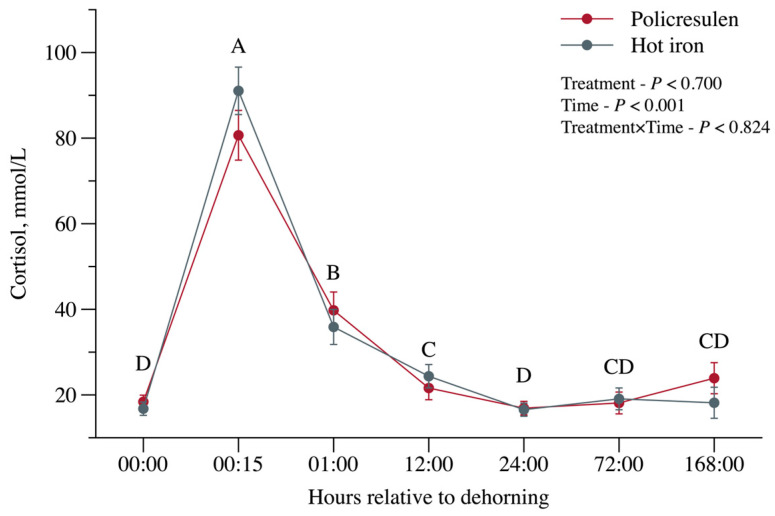
Serum cortisol concentrations in calves subjected to disbudding using either policresulen method or the hot iron method. Different capital letters indicate over time.

No interaction (*p* = 0.824) was observed between treatment and time for serum cortisol concentration. However, serum cortisol concentration increased (*p* < 0.001; Figure 4) 15 min after disbudding and returned to baseline within 24 h. No difference (*p* = 0.700) was found in serum cortisol concentrations between treatments.

**Figure 5:**
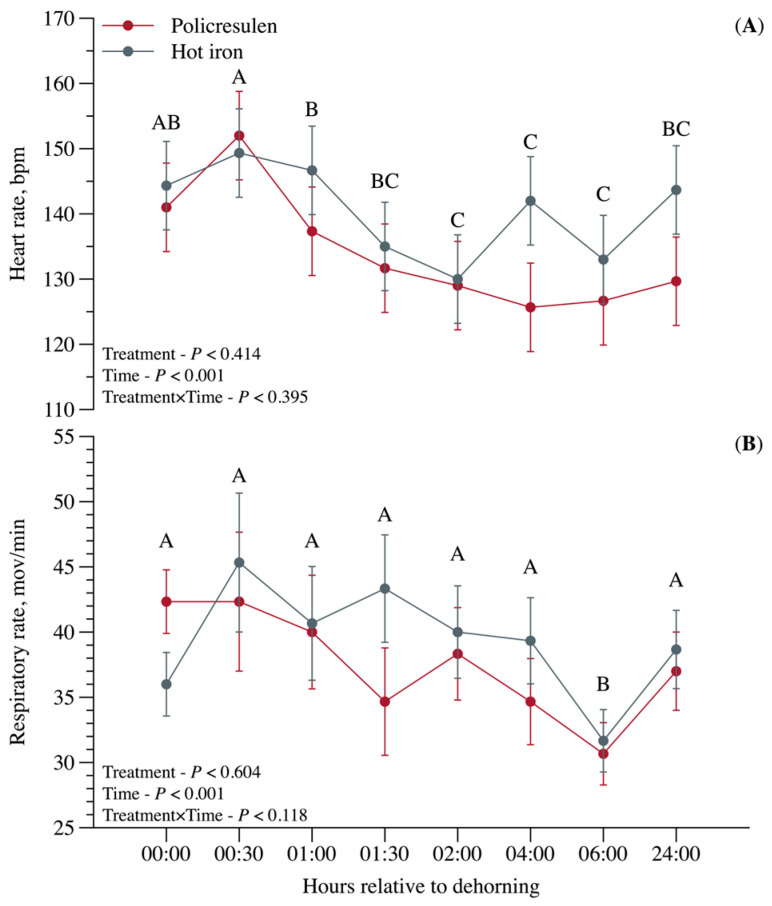
Heart rate (**A**) and respiratory rate (**B**) in calves subjected to disbudding using either policresulen method or the hot iron method. Different capital letters indicate difference for time.

No interaction effects (*p* > 0.05) were observed between treatment and time for heart and respiratory rates (Figure 5). Heart rate and respiratory rate did not differ between treatments (*p* > 0.05). However, both parameters fluctuated significantly in the first hours after the procedure (*p* < 0.001; Figure 5).

#### 3.1.3. Growth Performance

**Figure 6:**
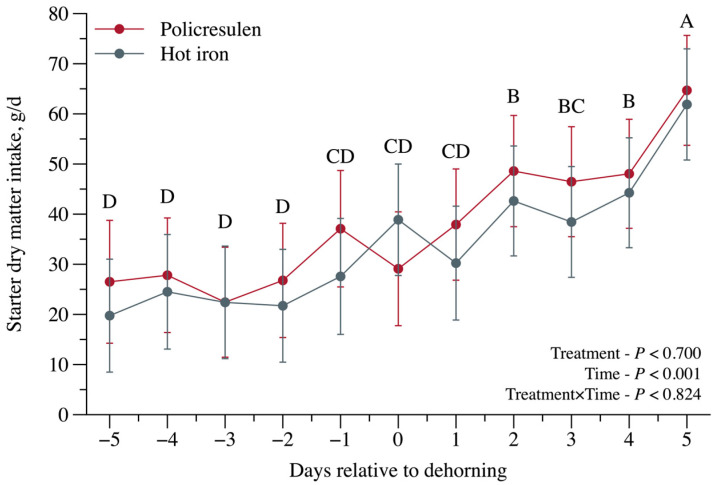
Starter dry matter intake in calves subjected to disbudding using either policresulen method or the hot iron method. Different capital letters indicate differences for time.

A time effect *(p* < 0.001; Figure 6) was observed for starter intake comparing days prior and after the disbudding procedure. Greater values were observed at 5 days after the procedure compared with previous days.

**Figure 7:**
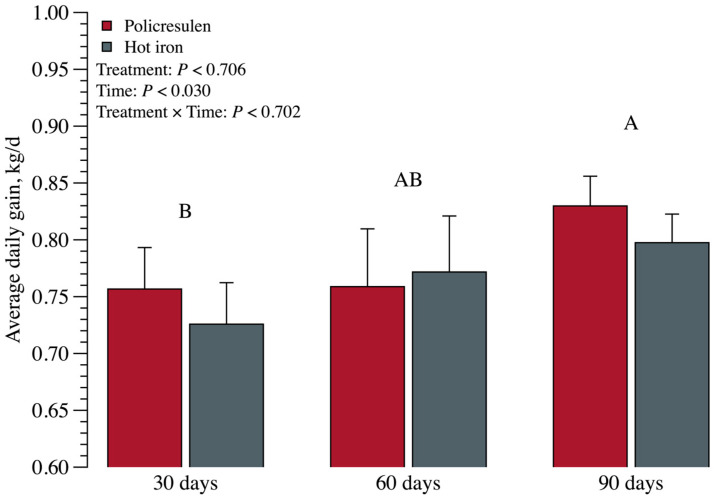
Average daily weight gain of calves at 30, 60, and 90 days after the disbudding procedure. Different capital letters indicate differences for time.

There was no interaction effect (*p* = 0.702; Figure 7) between treatment and time for the ADG at 30, 60, and 90 days after the disbudding procedure. However, a time effect (*p* < 0.030) was observed; ADG was higher at 90 days than at 30 days and no difference was observed between 30 and 60 days and between 60 and 90 days. No effect of the disbudding treatment (*p* = 0.076) was observed when evaluating ADG.

At the age of 12 months, however, no horn growth was observed in the calves treated with HID, while 9 out of 12 calves in the POD group showed horn growth. The regrown horn structures of the POD calves had a diameter of 2.76 ± 0.91 cm and a height of 3.40 ± 0.68 cm. 

### 3.2. Study 2—Dairy Consumer Perceptions of Calf Disbudding Methods

Of the 236 participants surveyed, 69% reported prior knowledge of the disbudding procedure and had experience or training in the agricultural sector. Those with an agricultural background were significantly more likely to be familiar with disbudding, 23.98 times more likely than those without this experience (*p* < 0.001; Table 1). They were also 13.74 times more likely to approve of the disbudding procedure (*p* < 0.001).

When comparing the two disbudding techniques, participants with agricultural experience were 3.8 times more likely to think that HID is easier to perform (*p* < 0.001) and 2.76 times more likely to think that HID is less invasive than POD (*p* < 0.001). In addition, these participants were also 2.76 times more likely to favor HID over POD as the method that justifies paying a premium price for dairy products (*p* < 0.001).

All participants with an agricultural background reported consuming dairy products (OR = 355.28; *p* < 0.001), while the two respondents who did not consume dairy products had a non-agricultural background.

Interestingly, the willingness to pay more for dairy products that came from farms that used animal–friendly disbudding methods was more common among participants without a farming background. The OR of willingness to pay a premium price was significantly lower among participants with farming experience (OR = 0.46; *p* < 0.001), suggesting that technical and practical considerations have a stronger influence in this group than animal welfare concerns.

## 4. Discussion

### 4.1. Study 1—Disbudding Techniques

Only the head shaking and the scratching of the horn region (with a limb or on objects) differed between the treatments. The combined use of local anesthetics and analgesics during disbudding has been shown to significantly reduce or even eliminate pain [1], which may explain the small behavioral differences between the groups in the present study. Both analgesics and horn nerve blocks were used to minimize pain, which likely contributed to the lack of differences in most behavioral and physiological parameters. Despite their importance, the use of anesthesia and analgesia in calf disbudding is still uncommon on many dairy farms [3].

Although POD is a combination of acids that causes corrosion of keratinized epithelium [13], HID has been observed to cause more open wounds with greater tissue exposure (as shown in Figure 2). The higher frequency of head shaking and scratching in the horn area in calves treated with HID probably indicates greater discomfort compared to calves treated with POD, as this treatment is less invasive and forms fewer open wounds [16]. Although complete epithelialization of the horn bud region was observed 10 weeks after the procedure in both treatments, calves in the POD group showed a greater decrease in horn bud circumference compared to the HID group, suggesting faster healing.

Although both treatments led to an increase in serum cortisol levels—an indicator of acute stress—levels returned to baseline within 1 h of disbudding. This rapid normalization can be attributed to the administration of local anesthetics and analgesics, which have been shown to reduce prolonged stress and pain responses [17]. The transient increase in cortisol levels in the first hour and the increased heart and respiratory rates observed in the first 30 min may be due to animal handling and physical restraint rather than the disbudding itself. Similar heart rate patterns were observed in dehorned calves with and without stunning [18], where it was found that stunned calves returned to their baseline values faster than unhandled calves.

Although this does not indicate a complete attenuation of treatment-specific pain, the immediate physiological changes observed in the present study due to the transient increase in cortisol, heart rate and respiratory rate are more likely related to the stress caused by the treatment and restraint. When compared study [19], the results showing an increase in heart rate during handling are similar, supporting our hypothesis that the rise in heart rate is linked to animal handling. However, if we consider not only the increase in heart rate during handling but also as an indicator of pain, calves that received cornual nerve block with lidocaine combined with oral anti-inflammatory (salicin) showed lower heart rates, highlighting the importance of using a combination of anesthetics and analgesics in calf disbudding techniques.

Feed intake increased over time in both groups, and no differences in ADG or TAG were observed prior to weaning. The comparable weight gain between calves disbudded with a hot iron and those not disbudded, with no significant difference, may indicate that the procedure itself had no direct effect on calf growth [5].

However, at 12 months of age, the horns grew back in 9 of 12 POD-treated calves, with horn structures measuring 2.76 ± 0.91 cm in diameter and 3.40 ± 0.68 cm in height, whereas no regrowth was observed in the HID group. It has been suggested that the age at which disbudding was performed (21 ± 2 days) may have contributed to this result, as the younger the animal, the less organized the keratinized epithelium [13]. The earlier the disbudding is performed in very young calves, the greater the sensitivity to pain, which may affect animal welfare [5]. Together with the fact that 75% of calves treated with POD later required surgical disbudding, the use of policresulen for disbudding was not effective as a non-surgical and less invasive alternative under the conditions of this study. Furthermore, a low number of experimental units in the study can be considered, with only 12 animals per treatment, which may hinder broader conclusions and results, with the possibility of different outcomes if replicated on a larger scale.

Given the recent introduction of policresulen for this purpose, further investigation is required to refine the protocol and evaluate its long-term efficacy in completely inhibiting horn bud development and avoiding the need for subsequent surgical intervention.

### 4.2. Study 2—Dairy Consumer Perceptions of Calf Disbudding Methods

The familiarity of individuals with agricultural experience or training with culling techniques emphasizes the importance of technical training in understanding common livestock management practices. Individuals with an agricultural background or practical experience tend to show a greater understanding of practices related to livestock management, such that they are more likely to agree with practices such as disbudding when justified by safety, management or productivity concerns [20]. The preference for HID among those with farming experience may be explained by their familiarity with the method, which is widely used on farms and, although unpleasant for the animal, is considered a reliable technique when performed properly [21]. The perception of HID as less invasive could be related to its technical feasibility and economic efficiency, although biological evidence shows that POD causes less extensive wounds [13]. In addition, farmers are often reluctant to introduce new alternatives in the absence of clear cost–benefit evidence [20].

The fact that all participants belonging to the agricultural sector in some way reported consuming dairy products may reflect their more positive perception of the production system. Professionals from the agricultural sector tend to have more confidence in the production processes and are generally less influenced by ethical or environmental concerns raised by the media or non-governmental organizations [22]. There is a large gap between producers and consumers, as concern for animal welfare and willingness to pay for welfare-certified products is accompanied by a limited understanding of production systems. This gap is largely due to the spatial and informational distance between urban consumers and farms, as well as the influence of the media and animal welfare organizations, which often highlight negative aspects of animal farming without providing a balanced picture [23]. As a result, consumer opinion is often shaped more by perceptions and misconceptions than by evidence-based knowledge. Anthropomorphism also influences public attitudes towards animal husbandry and emphasizes the need for effective scientific communication about animal welfare and the realities of husbandry systems [24].

In addition to farming background, demographic factors such as age, income and education also influence concern for animal welfare. Younger people, particularly those who are frequently exposed to social media, tend to show a greater awareness of animal welfare issues. Although concern is expressed across all income levels, people with higher incomes are generally more willing to pay premiums for animal welfare certified products. Similarly, higher levels of education are associated with a greater understanding of and concern for farming systems [20].

Together, these factors shape the profile of consumers who are interested in animal welfare. Despite studies on producers’ and consumers’ perspectives [24], research addressing the knowledge gap between agribusiness stakeholders and those outside the sector remains scarce. Differences in technical training, practical experience and less susceptibility to external influences contribute to the contrasting views. Agricultural professionals who work directly with animals tend to prioritize health, productivity and economic viability and are generally less influenced by media reports. In contrast, people who do not work in agriculture often rely on secondary sources of information and may form their opinions based on idealized portrayals or selective reporting [20].

In the present study, dairy consumers without farming experience showed a greater willingness to pay a premium price for products from farms that use welfare-friendly disbudding methods, especially POD, which they perceived as less invasive. This behavior can be attributed primarily to the urban population, which generally has little direct contact with agricultural production [18]. This behavior reflects consumers’ growing interest in animal welfare, although purchasing decisions may be based on perception rather than science [25]. The increasing sensitivity to practices that are considered “invasive” is related to the increasing distance from the reality of animal production and the influence of idealized representations of human–animal relationships [23].

This growing interest is an opportunity for the dairy sector to increase productivity while developing value-added products [24]. However, animal welfare is a complex, multidimensional concept that remains difficult to evaluate. Recent research has focused on the development of science-based animal welfare indicators and their inclusion in certification schemes [26].

The increasing demand for animal welfare-friendly dairy production represents both a challenge and an opportunity. While agribusiness professionals often take a technical and production-oriented perspective [22], urban consumers tend to form their opinions based on emotional reactions, media reports and social expectations [23]. Adopting transparent, science-based animal welfare practices can help reconcile these different viewpoints, increase competitiveness and improve the public image of the dairy industry. Therefore, the dissemination of clear, accessible and evidence-based information is crucial to close the knowledge gap and promote more informed consumer choices that are in line with sustainable production practices [24].

## 5. Conclusions

Disbudding with policresulen resulted in smaller wounds and faster healing compared to the conventional hot iron method. However, although policresulen may provide short-term benefits to calf welfare, it was not effective under the conditions of this study. Furthermore, a low number of experimental units in the study can be considered, with only 12 animals per treatment, which may hinder broader conclusions and results, with the possibility of different outcomes if replicated on a larger scale.

The contrasting views of those with some farming experience and those with no experience of disbudding methods highlight the influence of technical knowledge, practical experience and access to accurate information on perceptions of animal welfare. While those who work in agriculture tend to focus on effectiveness, feasibility and productivity, consumers—especially those who have no direct connection to the sector—are more likely to be influenced by ethical concerns, media coverage, and idealized notions of animal care. Nevertheless, they are interested in paying higher prices for products that are certified as animal welfare-friendly.

## Figures and Tables

**Table 1:** Participation of people related to the agricultural field in research on consumer perception of dairy products.

Itens	Answer	Odds ^a^	SE ^b^	*p*-Value
Prior knowledge of disbudding	Yes	23.98	0.4339	<0.001
Approval of disbudding	Yes	13.74	0.4969	<0.001
Perceived ease of performing each technique	HID ^c^	3.8	0.3017	<0.001
Perceived invasiveness	HID	2.76	0.3582	<0.001
Consumer of dairy products	Yes	355.28	>999.99	<0.001
Willingness to pay a premium for dairy products from farms that use welfare-friendly disbudding methods	Yes	0.46	0.3379	<0.001
Preference regarding wich disbudding method justifies paying a higher price	HID ^c^	2.76	0.3582	<0.001

^a^ Odds = Odds ratio. ^b^ SE = Standard error. ^c^ HID = hot iron disbudding.

## Data Availability

The original contributions presented in this study are included in the article. Further inquiries can be directed to the corresponding author.

## Abbreviations

The following abbreviations are used in this manuscript:

ADG	Average daily weight gain
POD	Policresulen disbudding
HID	Hot-iron disbudding
TAG	Total average gain
